# Evaluation of a Sensitive Visual Read Algorithm for Assessing 3R/4R Tau PET Images

**DOI:** 10.2967/jnumed.125.269685

**Published:** 2025-11

**Authors:** Ruben Smith, Valentina Garibotto, Douglas Hägerström, Jonas Jögi, Tomas Ohlsson, Olof Strandberg, Matteo Tonietto, Shorena Janelidze, Sebastian Palmqvist, Erik Stomrud, Gregory Klein, Oskar Hansson

**Affiliations:** 1 Clinical Memory Research Unit, Department of Clinical Sciences in Malmö, Lund University, Lund, Sweden;; 2 Memory Clinic, Skåne University Hospital, Malmö, Sweden;; 3 Division of Nuclear Medicine and Molecular Imaging, Diagnostic Department, University Hospitals of Geneva, Geneva, Switzerland;; 4 NIMTlab, Faculty of Medicine, University of Geneva, Geneva, Switzerland;; 5 Center for Biomedical Imaging, Geneva, Switzerland;; 6 Department of Clinical Neurophysiology, Skåne University Hospital, Lund, Sweden;; 7 Department of Clinical Physiology and Nuclear Medicine, Skåne University Hospital, Lund, Sweden;; 8 Department of Radiation Physics, Skåne University Hospital, Lund, Sweden; and; 9 F. Hoffmann-La Roche Ltd., Basel, Switzerland

**Keywords:** AD, tau PET, visual read, diagnostic accuracy, biomarkers

## Abstract

Developing and validating sensitive visual read algorithms for assessing Alzheimer disease–related tau in tau PET imaging is imperative, considering the implementation of the methodology in clinical practice and trials. Our aim was to compare 2 visual read algorithms for tau PET images to semiquantitative measurements and plasma phospho-tau 217 (p-tau217) status. **Methods:** In total, 1,654 participants were consecutively recruited from secondary memory clinics in southern Sweden as part of the prospective BioFINDER-2 cohort study (May 2017–September 2023). All participants underwent [^18^F]RO948 scans, and 37 participants underwent an additional [^18^F]flortaucipir scan. PET scans were read visually in accordance with the BioFINDER visual read (BF-VR) protocol and the established visual read method for [^18^F]flortaucipir (FTP-VR). Comparative analyses were conducted with semiquantitative SUV ratios (SUVRs) in the entorhinal cortex (ERC) and a temporal meta-region of interest and with plasma p-tau217 status. The primary endpoints of the study included the accuracy of visual read algorithms in detecting increases in semiquantitative SUVRs and p-tau217. Secondary outcomes included the intrarater and interrater reliabilities of the BF-VR. **Results:** Both visual read methods exhibited strong concordance with semiquantitative SUVRs. However, the BF-VR method demonstrated superior accuracy for tau in the ERC (93.9%; 95% CI, 92.6%–95.0%) compared with the FTP-VR method (89.9%; 95% CI, 88.3%–91.3%; *P* < 0.0001). The BF-VRs displayed lower accuracy when detecting tau in the temporal meta-region of interest (91.8%; 95% CI, 90.4%–93.1%) compared with the FTP-VRs (94.7%; 95% CI, 93.6%–95.8%; *P* = 0.002). Further, the BF-VRs exhibited similar accuracy (0.866; 95% CI, 0.847–0.884) to the FTP-VRs (0.857; 95% CI, 0.837–0.875; *P* = 0.54) for detection of p-tau217 abnormality. The interrater reliability of the BF-VR algorithm was excellent (weighted Cohen κ, 0.87; range, 0.82–0.93), and intrarater reliability was almost perfect (Cohen κ, 0.94; range, 0.89–0.98). The concordance of the BF-VR assessments of [^18^F]RO948 and [^18^F]flortaucipir images was excellent (Cohen κ, 0.94; range, 0.86–1.00). **Conclusion:** Visual reads offer a straightforward method for evaluating tau PET scans. The BF-VR algorithm provides a more accurate algorithm for detecting tau uptake in the ERC compared with the established FTP-VR algorithm and exhibits similar performance when using [^18^F]RO948 and [^18^F]flortaucipir images. The BF-VR algorithm further showed excellent interrater and intrarater reliabilities.

## Visual Abstract

**Figure:**
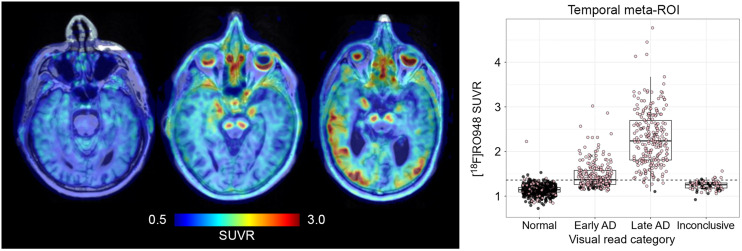

With the development of PET radiotracers for tau pathology (1), a hallmark of Alzheimer disease (AD) (2), tau pathology can be tracked in vivo. Tau PET tracers bind to tau pathologies in vitro (3), and tau PET uptake in vivo correlates to AD-type 3R/4R tauopathies postmortem (4–7). The finding that [^18^F]flortaucipir visual reads could reliably detect tauopathies in Braak stages V and VI (4) (widespread neocortical tau pathology) led to the approval of [^18^F]flortaucipir by the Food and Drug Administration as a biomarker for supporting a diagnosis of AD (8).

There are now several proposed algorithms available for visual reads of tau PET scans (4,9,10). The only visual read algorithm approved by the Food and Drug Administration is the [^18^F]flortaucipir visual read (FTP-VR) protocol developed by Fleisher et al. (4). It utilizes the regional uptake in the posterolateral temporal or occipital regions as a minimal requirement for positivity (moderate AD tau pattern). Increased neocortical tau PET uptake isolated to the mesial temporal, anterolateral temporal, or frontal regions was considered not consistent with AD and seen as a negative AD tau pattern. This visual assessment correlates well to late-stage AD neuropathology (Braak stages V and VI) (4) but shows a poorer association to earlier stage AD pathology. More recently, Seibyl et al. (9) introduced a visual read algorithm for assessing [^18^F]MK-6240 PET scans. This algorithm allowed for a positive visual read of the tau PET scan with uptake in the bilateral medial temporal lobes (positivity in at least 2 regions required for a determination of tau positivity) (9). In this study, we used [^18^F]RO948 for tau PET scanning. Its structure is highly related to that of [^18^F]flortaucipir (1), although there are differences in off-target binding between tracers. [^18^F]RO948 scans show lower binding in the choroid plexus and basal ganglia (11) and higher binding in the meninges and bone (12). Although the FTP-VR approach enhances specificity for tau pathology, it may reduce sensitivity, potentially leading to an underdiagnosis of early AD pathology. Notably, tau pathology commonly starts to accumulate at an early age in the medial temporal lobes (13) and spreads with amyloid-β (Aβ) positivity from the medial temporal lobes, through the temporal lobes, and throughout the neocortex (2,13,14). Recently published data indicate that medial temporal lobe positivity on tau PET confers an increased risk of future cognitive decline and conversion to mild cognitive impairment in cognitively unimpaired persons (15). Consequently, Aβ positivity and tau PET uptake restricted to the medial temporal areas has been suggested as an early stage (stage B) of AD in the revised criteria for AD (16), stressing the importance of identifying this early stage of AD in visual read algorithms.

We recently showed that the BioFINDER visual read (BF-VR) algorithm has an added clinical value for diagnosis, treatment, and diagnostic certainty in addition to an extensive diagnostic work-up in a memory clinic setting (10). Here, we aim to compare the BF-VR algorithm to the established FTP-VR method, determine the accuracy of the visual reads compared with semiquantitative assessments of tau PET using SUV ratios (SUVRs), compare how well visual reads and semiquantitative measures correlate with positivity in an independent AD biomarker (phospho-tau 217 [p-tau217]), assess the interrater and intrarater reliabilities of the BF-VR algorithm, and evaluate how well the BF-VR algorithm translates to scans with the tau PET tracer [^18^F]flortaucipir.

## MATERIALS AND METHODS

### Participants

In total, 1,654 participants of the BioFINDER-2 study (NCT03174938), recruited between April 2017 and September 2023, were included in the analysis. Details on participant characteristics and recruitment are provided in Table 1 and in the supplemental materials, available at http://jnm.snmjournals.org (17,18).

**TABLE 1.** Participant Demographics

						*P*				
Characteristic	All	Controls	SCD	MCI	Dementia	Dementia vs. MCI	Dementia vs. controls	Controls vs. SCD	MCI vs. SCD	MCI vs. controls
*n* *	1654	578	253	398	389					
Age (y)	68.4 ± 12.1	63.5 ± 15.6	67.5 ± 9.4	71.5 ± 7.9	73.2 ± 7.5	0.0014	<0.0001	0.017	<0.0001	<0.0001
Sex							0.003	0.027	0.045	<0.0001
Female	817 (49.4)	333 (57.6)	124 (49.0)	162 (40.7)	185 (47.6)					
Male	837 (50.6)	245 (42.4)	129 (51.0)	236 (59.3)	204 (52.4)					
Aβ-positive^†^	754 (46.3)	114 (19.7)	113 (44.7)	222 (55.8)	300 (77.1)	<0.0001	<0.0001	<0.0001	0.007	<0.0001
MMSE	26.7 ± 4.0	29.0 ± 1.3	28.6 ± 1.5	27.1 ± 2.0	21.7 ± 4.5	<0.0001	<0.0001	0.007	<0.0001	<0.0001
[^18^F]RO948 in ERC	1.36 ± 0.42	1.14 ± 0.17	1.25 ± 0.30	1.41 ± 0.41	1.70 ± 0.51	<0.0001	<0.0001	<0.0001	<0.0001	<0.0001
[^18^F]RO948 in temporal meta-ROI	1.36 ± 0.48	1.15 ± 0.11	1.22 ± 0.25	1.35 ± 0.39	1.77 ± 0.72	<0.0001	<0.0001	<0.0001	<0.0001	<0.0001
Plasma p-tau217 (pg/mL)^‡^	0.324 ± 0.310	0.189 ± 0.122	0.232 ± 0.152	0.337 ± 0.326	0.562 ± 0.298	<0.0001	<0.0001	0.002	<0.0001	<0.0001
BF-VR result^§^						<0.001	<0.001			
Normal	1092	524	190	234	121					
Inconclusive	89	23	23	21	21					
Early	233	28	26	78	95					
Late	240	3	14	65	152					

* Data missing for 36 participants.

† Data missing for 26 participants.

‡ Data missing for 247 participants (90 controls, 41 SCD, 41 MCI, and 47 with dementia).

§ *P* <0.001 for participants with dementia vs. mild cognitive impairment.

SCD = subjective cognitive decline; MCI = mild cognitive impairment; MMSE = Mini-Mental State Examination.

Data for [^18^F]RO948 expressed in SUVR ± SD; all other data expressed as mean ± SD or number and percentage.

The study was conducted in accordance with the Declaration of Helsinki and approved by the regional review board for human research ethics at Lund University. Written informed consent was provided by all participants at the time of study inclusion.

### Image Acquisition and Processing

[^18^F]RO948 PET data were acquired on digital Discovery MI PET/CT scanners (GE HealthCare). List-mode image data were collected 70–90 min after injection of approximately 370 MBq of [^18^F]RO948. Details on PET scan reconstruction and image processing are provided in the supplemental materials (18,19).

The BF-VR method was performed using a rainbow color scale, with the reference inferior cerebellar cortex set at the blue-to-cyan color shift. Images were coregistered to low-dose attenuation-correction CT scans for better anatomic detail and to help distinguish entorhinal cortex (ERC) signal from the off-target signal derived from neighboring meninges and bone structures. Evaluations were performed by 2 raters who reached a joint decision on how to categorize each image, as previously described (10). Visual reads were categorized as one of the following: normal image (no discernible [^18^F]RO948 retention); retention of [^18^F]RO948 confined to the temporal lobes but allowing positivity with unilateral medial temporal lobe uptake; widespread retention of [^18^F]RO948, reaching into the parietal, occipital, or frontal lobes; and inconclusive scan. In analyses with a dichotomous negative and positive tau PET visual read, inconclusive visual reads were considered negative (i.e., not having a typical AD pattern). Images were read as soon as they became available for the duration of the study. For interrater reliability, 141 randomly selected images were read by an independent rater, who received an introduction to the algorithm and was trained on a training set of [^18^F]RO948 images before performing any ratings for this study. To determine the intrarater reliability, 100 randomly selected images were reread by 1 rater after all study images were acquired. In a sensitivity analysis, another 100 scans were reread to compare the outcome of the interrater read to the read conducted by the 1 rater. These results are presented in Supplemental Figure 1.

FTP-VR algorithm images were processed using methods previously described (4). In brief, images were processed using an in-house–developed pipeline that defined uptake in the cerebellar cortex. SUVR images were created and scaled to 0–3.3, using a color scale with a rapid shift in color (yellow to red) at 50% of the scale, corresponding to an SUVR of 1.65. Images were read over a 2-wk period after the initial BF-VR reads by a single rater, masked to clinical information, and classified into 1 of the 3 categories: no increased neocortical activity or increased neocortical activity isolated to the mesial temporal, anterolateral temporal, or frontal regions (4); moderate AD pattern with increased signal in the posterolateral or occipital cortex; or an advanced AD pattern with widespread neocortical uptake.

Thirty-seven participants further participated in a head-to-head study in which [^18^F]flortaucipir tau PET scans were performed within 36 ± 35 d from the [^18^F]RO948 PET scan, as previously described (11). The head-to-head data were visually read by the same single reader on 2 separate days, one week apart.

### Cerebrospinal Fluid (CSF) and Blood Sampling

Details on the handling of CSF and blood samples are provided in the supplemental materials (20–22).

### Statistical Analyses

Statistical analyses details are provided in the supplemental materials (23).

## RESULTS

### Participants

In total, 1,654 participants in the Swedish BioFINDER-2 study were included in the analysis. Participant characteristics are summarized in Table 1. Participants had a mean age of 68.4 ± 12.1 y, and 754 (46.3%) of 1,628 were Aβ-positive (details provided in the supplemental materials). All participants underwent a baseline [^18^F]RO948 tau PET scan. Of the 1,654 participants, 1,618 had data on baseline cognitive status, subdivided into cognitively normal, subjective cognitive decline, mild cognitive impairment, or dementia. Participants with subjective cognitive decline or objective memory impairment were older, had a higher prevalence of Aβ positivity, had higher levels of tau PET retention, and had plasma p-tau217 levels (Table 1).

### Visual Reads and Semiquantitative Measures

**FIGURE 1.**
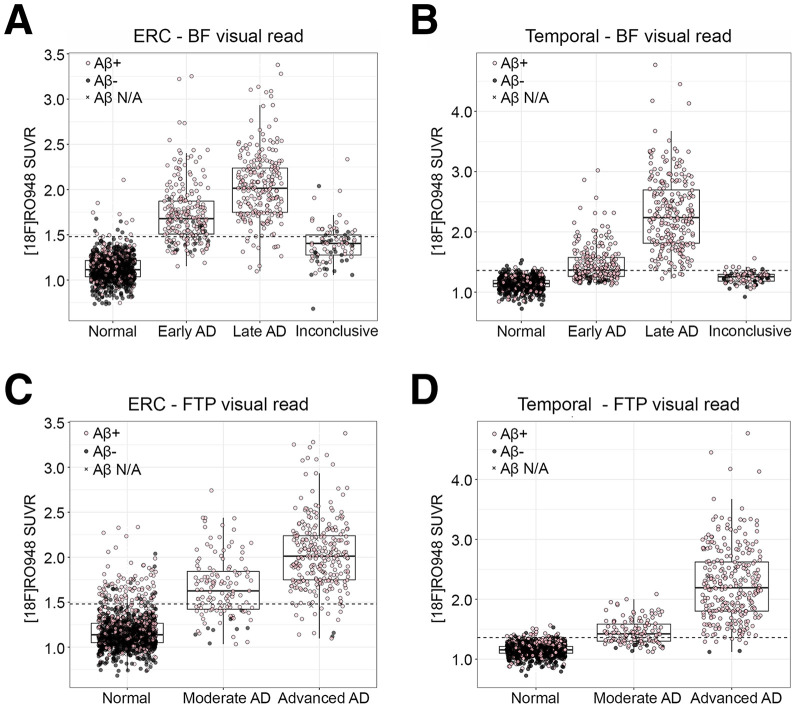
Comparison of visual read algorithms with semiquantitative measurements. SUVRs in ERC (A) and temporal meta-ROI (B) in BF-VR categories and ERC (C) and temporal meta-ROI (D) in different FTP-VR categories. Pink dots represent Aβ-positive participants, and black dots represent Aβ-negative participants. Dotted line represents cutoff for quantitative tau positivity (1.48 in ERC and 1.36 in temporal meta-ROI). N/A = not available.

Most of the tau PET images read using the BF-VR method were categorized as normal (*n* = 1,092; 66.0%), followed by late AD pattern (*n* = 240; 14.5%), early AD pattern (*n* = 233; 14.1%), or inconclusive (*n* = 89; 5.3%), as previously described (10). Processed data for the FTP-VR algorithm were available for 1,650 participants, of whom 1,247 (75.6%) had a normal read, 129 (7.8%) had a moderate AD pattern, and 274 (16.6%) had an advanced AD pattern. Positive visual reads were associated with an increased tau PET uptake in semiquantitative SUVR measures (*t* test; *P* < 0.0001 for all comparisons; Figs. 1A–1D). Using the 2 visual read algorithms, we found that 92.7% of reads were concordant and 7.3% were discordant, with a statistically significant difference between methods (McNemar test; *P* < 0.0001). Of the 110 discordant cases, 86% (95) were read as positive with the BF-VR method and as negative with the FTP-VR method; 14% (25) were read as negative with the BF-VR method and as positive with the FTP-VR method.

**FIGURE 2.**
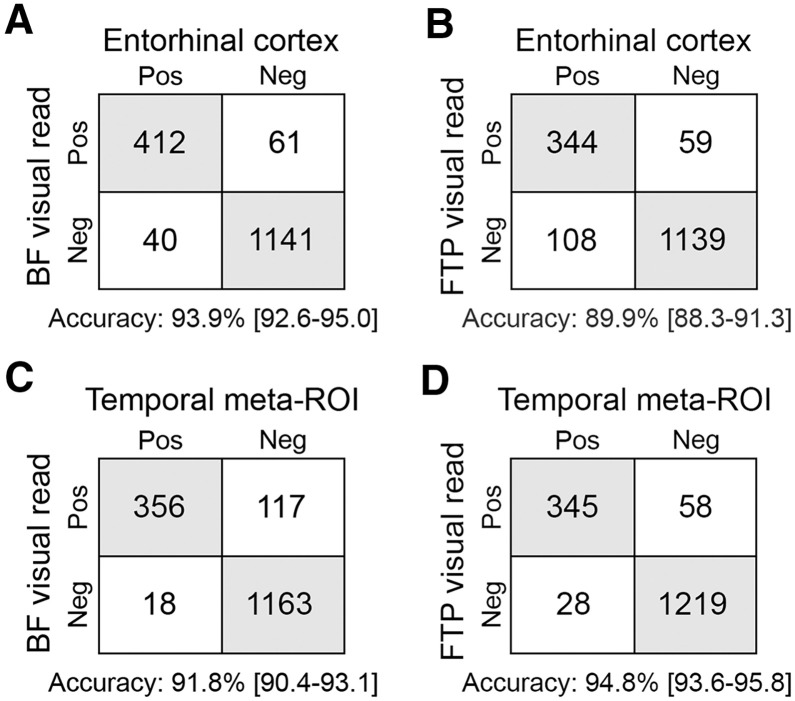
Concordance between SUVRs in entorhinal cortex and BF-VR (A) and FTP-VR (B). Concordance between SUVRs in temporal meta-ROI BF-VR (C) and FTP-VR (D). Accuracies for visual reads in determining abnormal semiquantitative values, with 95% CI in square brackets. Neg = negative; Pos = positive.

When compared with quantitative [^18^F]RO948 results, we found that BF-VRs were concordant with quantitative SUVR in the ERC in 1,549 of 1,650 cases (93.9%; 95% CI, 92.6%–95.0%; *P* < 0.0001 compared with FTP-VRs) and concordant in the temporal meta-region of interest (ROI) in 1,516 of 1,650 cases (91.8%; 95% CI, 90.4%–93.1%; *P* = 0.002 compared with FTP-VRs). Using the FTP-VR algorithm, concordance was lower in the ERC with 1,483 of 1,650 cases (89.9%; 95% CI, 88.3%–91.3%) but higher in the later temporal meta-ROI (1564 of 1650 cases; 94.7%; 95% CI, 93.6%–95.8%) (Figs. 2A–2D; Table 2). Results for a larger neocortical ROI are included in the supplementary information (Supplemental Fig. 2; Supplemental Table 1).

**TABLE 2.** Accuracy, PPV, and NPV for Visual Read Algorithms Compared with SUVRs and Plasma p-tau217

Variable	Accuracy	PPV	NPV
Comparison with tau PET SUVRs			
BF-VR vs. ERC SUVR	0.939 (0.926–0.95)	0.871 (0.837–0.9)	0.966 (0.954–0.976)
BF-VR vs. temporal meta-ROI SUVR	0.918 (0.904–0.931)	0.753 (0.711–0.791)	0.985 (0.976–0.991)
FTP-VR vs. ERC SUVR	0.899 (0.883–0.913)	0.854 (0.815–0.887)	0.913 (0.896–0.928)
FTP-VR vs. temporal meta-ROI SUVR	0.948 (0.936–0.958)	0.856 (0.818–0.889)	0.978 (0.968–0.985)
Comparison with p-tau217			
BF-VR vs. p–tau217	0.866 (0.847–0.884)	0.892 (0.857–0.92)	0.856 (0.833–0.877)
FTP-VR vs. p-tau217	0.857 (0.837–0.875)	0.936 (0.904–0.959)	0.831 (0.807–0.853)
ERC SUVR vs. p-tau217	0.848 (0.828–0.866)	0.872 (0.834–0.904)	0.839 (0.815–0.861)

PPV = positive predictive value; NPV = negative predictive value.

Values within brackets represent 95% CI.

Comparing the BF-VRs to semiquantitative results separated by Aβ and cognitive statuses showed, as expected, an increased prevalence of positive visual reads and pathologic semiquantitative values in the more-impaired groups compared with controls and participants with subjective cognitive decline (Supplemental Fig. 3). Nearly 95% of participants with positive (defined as early or late AD pattern) visual reads with the BF-VR algorithm had a pathologic CSF Aβ_42_/Aβ_40_ ratio. The majority of normal visual reads corresponded to a normal SUVR in the ERC (98.6%) and in the temporal meta-ROI (99.0%). The negative predictive value for the BF-VR was 96.6% in the ERC and 98.5% in the temporal meta-ROI (Table 2). For inconclusive reads, 71.9% had normal SUVRs in the ERC and 92.1% in the temporal meta-ROI (Figs. 1A and 1B). Data showing a combination of the 2 visual read algorithms are shown in Supplemental Figure 4.

### Visual Reads and Plasma p-tau217

**FIGURE 3.**
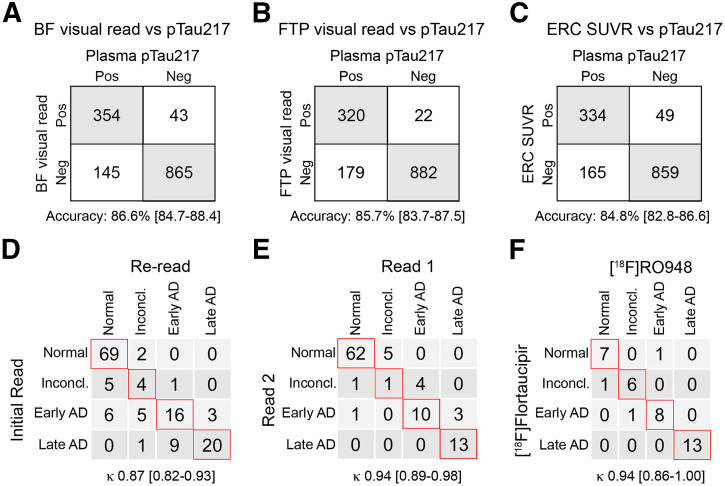
Comparison of visual read algorithms to plasma p-tau217 and interrater, intrarater, and intertracer concordance. Accuracies for BF-VRs (A), FTP-VRs (B), and ERC SUVRs (C) in determining pathologic plasma p-tau217. Number of included participants is 1,407 (A and C) and 1,403 (B), since only participants with available data for all biomarkers (plasma p-tau217, visual read and SUVRs from ERC, and temporal meta-ROI) were included in analysis. (D) Interrater agreement for 141 randomly selected tau PET scans. (E) Intrarater agreement is shown for 100 reread scans. (F) Concordance of visual read algorithm when transferred to related tau PET tracer [^18^F]flortaucipir in head-to-head dataset, with available data for both radiotracers (*n* = 37). κ represents Cohen weighted κ values. Numbers in square brackets represent 95% CI. Neg = negative; Pos = positive.

Accuracies were calculated for agreement with p-tau217 for both visual reads and for the ERC SUVR measurement. Because p-tau217 is an early biomarker of AD, and the temporal meta-ROI is a later-stage imaging biomarker, we included only the ERC in this analysis. The number of concordantly classified cases was 1,215 of 1,403 for the BF-VR algorithm, resulting in an accuracy with p-tau217 status of 0.866 (95% CI, 0.847–0.884). With the FTP-VR algorithm, 1,202 of 1,403 cases were concordant (accuracy, 0.857; 95% CI, 0.837–0.875), and 1,189 of 1,403 cases were concordant with the ERC SUVR (accuracy of 0.847; 95% CI, 0.828–0.866) (Figs. 3A–3C). No statistically significant differences were found among accuracies.

### Interrater and Intrarater Reliability of BF-VR Algorithm and Translatability to [^18^F]Flortaucipir Scans

To determine interrater reliability, an independent rater assessed 141 randomly selected tau PET scans and compared the results of the visual read algorithm with the initial read. The interrater reliability was excellent (weighted Cohen κ, 0.87; 95% CI, 0.82–0.93; Fig. 3D). To determine intrarater reliability, 100 tau PET scans were reread with an almost-perfect agreement (weighted Cohen κ, 0.94; 95% CI, 0.89–0.98; Fig. 3E).

To assess the performance of the visual read algorithm, established using [^18^F]RO948 images, in scans with the more widely available tau PET tracer [^18^F]flortaucipir, we visually assessed scans from the 37 participants who participated in a head-to-head study of [^18^F]RO948 and [^18^F]flortaucipir (11). Visual read results using the BioFINDER algorithm for both tracers showed an almost perfect concordance (weighted Cohen κ, 0.94; 95% CI, 0.86–1.00; Fig. 3F).

## DISCUSSION

In this study, we aimed to compare the BF-VR algorithm with the established FTP-VR protocol and semiquantitative measurements of tau pathology and to determine the visual read algorithm agreement with pathologic plasma p-tau217 status. As tau PET scans are increasingly used in clinical settings, and visual read remains the approved method for interpreting scan outcomes, it is crucial to determine the accuracy of these methods for detecting tau pathology associated with AD.

Several visual read algorithms have been previously published for [^18^F]MK-6240 (9,24) and [^18^F]flortaucipir (4,25,26). The approved FTP-VR method considers the tau PET signal in the posterior lateral temporal lobes, disregarding isolated pathology in the anterior and medial parts of the temporal lobes (4). The BF-VR method presented here allows for visual read positivity in just 1 medial temporal lobe, thereby enhancing sensitivity but also introducing a potential risk of false-positive results from off-target binding. As anticipated, the accuracy of the BF-VR algorithm in detecting increases in semiquantitative SUVRs was higher than that of the FTP-VR method in the early ERC ROI but lower in the later temporal meta-ROI. However, there were no differences seen in the accuracy of the BF-VR algorithm compared with the FTP-VR method and to SUVRs in the ERC for detecting a pathologic plasma p-tau217 status.

The accuracy for determining a pathologic SUVR using the BF-VR method was 93.9% for the ERC and 91.8% for the temporal meta-ROI. The lower accuracy of the temporal meta-ROI can be attributed to a larger proportion of early AD pattern visual reads not meeting the positivity cutoff for semiquantitative measures in this region with later tau accumulation. Notably, the number of false-negative reads was limited, particularly when using the larger temporal meta-ROI as the outcome, as indicated by high negative predictive values (96.6% for the ERC and 98.5% for the temporal meta-ROI). As expected, the positive predictive values were lower (87.1% with the ERC as the outcome and 75.3% for the temporal meta-ROI), primarily because some participants with positive visual reads did not reach the cutoff for semiquantitative positivity.

The discrepancy between positivity in visual reads and SUVRs raises questions about the correct gold standard for determining tau PET positivity. Although semiquantitative measurements may underestimate tau pathology because of ROI limitations, visual inspection, not constrained by predefined brain region delineations, can detect more localized positivity. However, approximately 5% of cases resulted in inconclusive visual reads, primarily attributable to off-target binding in adjacent meninges or bone structures, combined with equivocal retention in the medial temporal lobe cortex. Among scans read as positive, all images identified as having a late AD pattern were Aβ positive, and 92.6% of participants classified as having an early AD pattern were Aβ positive, indicating that the visual read algorithm largely identifies early true tau pathology in Aβ-positive individuals.

To assess the reproducibility of the visual read algorithm, 100 scans were reread by the same experienced rater, revealing an almost-perfect (23) intrarater reliability (weighted Cohen κ, 0.94). Additionally, interrater reliability was assessed by a second rater who rated 141 scans after a brief training in the visual read algorithm, yielding excellent interrater reliability (weighted Cohen κ, 0.87) despite the short training period. Interrater reliability was similar to previously reported reliability reported for [^18^F]MK-6240 (9) and marginally lower than for another visual read algorithm for [^18^F]flortaucipir (27), although intrarater reliability was similar.

Compared with [^18^F]flortaucipir, [^18^F]RO948 exhibits lower off-target binding to the basal ganglia and the choroid plexus (11). Nonetheless, the performance of the proposed visual read algorithm was similar with both tracers, demonstrating excellent intertracer agreement (weighted Cohen κ, 0.94). Some of the disagreement seen between the radiotracers can be attributed to 5 participants with semantic dementia who were included in the head-to-head dataset, where the off-target binding was stronger with [^18^F]flortaucipir than with [^18^F]RO948 (18). The higher choroid plexus signal in [^18^F]flortaucipir could theoretically interfere with the interpretation of the binding in the medial temporal lobes, but we found that the choroid plexus binding could be disregarded in our visual reads because of the more dorsolateral location compared with the early medial temporal cortex signal (Supplemental Fig. 5). Of the 5 participants in the head-to-head dataset with tau restricted to the medial temporal lobes, the images of 4 were read similarly with both tracers, and 1 was discordant (positive in the [^18^F]RO948 scan and negative in the [^18^F]flortaucipir scan).

The primary limitation of this study was the absence of neuropathologic verification of tau pathology in the participants, precluding a gold standard reference for the detected tau PET signal. To mitigate this limitation, we included plasma p-tau217 as a PET-independent marker of AD pathology (22,28,29). It is well established that p-tau217 is an earlier biomarker than tau PET (29,30); because of this, the accuracies for both tau PET visual read biomarkers were reduced. Future studies should address the sensitivity and specificity of the visual read methods for detecting neuropathologic tau. A second limitation was that the study was designed with a 2-reader joint-decision read for the BF-VR algorithm, whereas only one reader interpreted images with the FTP-VR protocol. To control whether a joint-decision read differed from a single-rater read, 100 scans from the BF-VR algorithm were reread by a single reader, showing a high concordance to the initial read (Cohen κ, 0.95; Supplemental Fig. 1). Another limitation was that head-to-head imaging of [^18^F]RO948 and [^18^F]flortaucipir was performed in a limited number of participants, and performance of the visual read algorithm in [^18^F]flortaucipir scans will have to be confirmed in an independent, larger dataset.

## CONCLUSION

The BF-VR algorithm provides a straightforward method for assessing tau pathology in the brain. It exhibits high accuracy in determining semiquantitative results and high negative predictive values for both early- and late-stage tau. Furthermore, the algorithm demonstrates excellent interrater reliability, intrarater reliability, and concordance between PET tracers, suggesting it may be applicable also to visual reads of [^18^F]flortaucipir scans.

## DISCLOSURE

This study was supported by the European Research Council (ADG-101096455), Alzheimer’s Association (ZEN24-1069572, SG-23–1061717), GHR Foundation, Swedish Research Council (2022-00775), ERA PerMed (ERAPERMED2021-184), Knut and Alice Wallenberg Foundation (2022-0231), Strategic Research Area MultiPark (Multidisciplinary Research in Parkinson Disease) at Lund University, the Swedish Alzheimer Foundation (AF-980907; AF-939981), the Swedish Brain Foundation (FO2021-0293), The Parkinson Foundation of Sweden (1280/20), the Cure Alzheimer’s Fund, the Konung Gustaf V:s och Drottning Victorias Frimurarestiftelse, the Skåne University Hospital Foundation (2020-O000028), Regionalt Forskningsstöd (2020-0314; 2021-1013), the Kockska Foundation, Bundy Academy, and the Swedish federal government under the ALF agreement (2022-Projekt0080; 2020-YF0020). The funding sources had no role in design and conduct of the study; collection, management, analysis, and interpretation of the data; preparation, review, or approval of the manuscript; and decision to submit the manuscript for publication. The precursor of [^18^F]RO948 was provided by Roche. Ruben Smith has received consultancy/speaker fees from Eli Lilly, Novo Nordisk, Roche, and Triolab. Matteo Tonietto and Gregory Klein are full-time employees of Hoffman-La Roche Ltd. In the past year, Tomas Ohlsson has received consultancy fees from Spago Nanomedical. Oskar Hansson is an employee of Lilly and Lund University. Sebastian Palmqvist has acquired research support (for the institution) from ki elements/ADDF and Avid; in the past 2 y, he has received consultancy/speaker fees from Bioartic, Biogen, Esai, Lilly, and Roche. Valentina Garibotto received research support through her institution and speaker fees from Siemens Healthineers, GE HealthCare, Janssen, and Novo Nordisk. No other potential conflict of interest relevant to this article was reported.

## Notes

KEY POINTS **QUESTION:** Can a new tau PET visual read algorithm effectively identify early changes associated with AD? **PERTINENT FINDINGS:** In a prospective, secondary memory clinic cohort study involving 1,654 patients with [^18^F]RO948 PET imaging, we observed that incorporating the medial temporal lobes into a visual read algorithm enhanced its accuracy in detecting early AD changes compared with the existing reading protocol. Moreover, the new visual read algorithm demonstrates excellent intrarater and interrater reliability and performs comparably well with widely available [^18^F]flortaucipir PET scans. **IMPLICATIONS FOR PATIENT CARE:** Employing a tau PET visual read algorithm that allows for a positive reading based on medial temporal lobe uptake enhances accuracy in the detection of early-AD changes.
